# A scoping review of patient and public involvement in empirical stroke research

**DOI:** 10.1177/17474930241262638

**Published:** 2024-07-31

**Authors:** Paula da Cruz Peniche, Christina Danielli Coelho de Morais Faria, Patricia Hall, Caitriona Fingleton, Louise McPhillips, Rebecca Gaetz, Aaron Roche, Laura McCann, Padraig O’Beaglaoich, Diarmuid Murphy, Julianne Hickey, Olive Lennon

**Affiliations:** 1 Postgraduate Program in Rehabilitation Science, Universidade Federal de Minas Gerais, Belo Horizonte, Brazil; 2 Department of Physical Therapy, Universidade Federal de Minas Gerais (UFMG), Belo Horizonte, Brazil; 3 School of Public Health, Physiotherapy and Sports Science, University College Dublin, Dublin, Ireland; 4 Improving Pathways for Acute Stroke and Rehabilitation (iPASTAR) Collaborative Doctoral Award and PPI Panel, Royal College of Surgeons, Dublin, Ireland; 5 National Rehabilitation Hospital, Dublin, Ireland

**Keywords:** Patient and public involvement, patient engagement, patient participation, stakeholder involvement, stroke, review

## Abstract

**Background:**

Impactful, evidence-based solutions in surveillance, prevention, acute care, and rehabilitation for stroke survivors are required to address the high global burden of stroke. Patient and public involvement (PPI), where patients, their families, and the public are actively involved as research partners, enhances the relevance, credibility, and impact of stroke-related research.

**Aims:**

This scoping review, adhering to the Preferred Reporting Items for Systematic Reviews and Meta-Analysis (PRISMA) Scoping Review guidelines, aims to identify and summarize how PPI is currently implemented and reported in empirical stroke research using a participatory approach.

**Summary of review:**

A comprehensive search strategy was developed and implemented across Medline, CINAHL, EMBASE, PsynchINFO, and Cochrane electronic databases, supplemented by gray literature searches. Empirical stroke research articles in the English language, published from 2014 up to 2023, and documenting PPI activity were included. Of the 18,143 original articles identified, 2824 full-text manuscripts matching from this time window were screened. Only 2% (n = 72) of these directly reported embedded PPI activity in empirical research. The majority were qualitative in design (60%) and conducted in high-income countries (96%). Only one included study originated from a developing country, where the burden of stroke is highest. Most studies (94%) provided some information about the activities carried out with their PPI partners, mainly centered on the study design (57%) and management (64%), with only 4% of studies integrating PPI across all research cycle phases from funding application to dissemination. When studies were examined for compliance with the Guidance for Reporting Involvement of Patients and the Public (GRIPP) short-form checklist, only 11% of included studies were 100% compliant. Twenty-one studies (29%) reported barriers and facilitators to including PPI in stroke research. Organization, authentic partnership, and experienced PPI representatives were common facilitators and identified barriers reflected concerns around adequate funding, time required, and diversity in perspectives. A positive reporting bias for PPI impact was observed, summarized as keeping the patient perspective central to the research process, improved care of study participants, validation of study findings, and improved communication/lay-summaries of complex research concepts.

**Conclusions:**

PPI is underutilized and inconsistently reported in current empirical stroke research. PPI must become more widely adopted, notably in low- and middle-income countries. Consensus-driven standards for inclusion of PPI by funding organizations and publishers are required to support its widespread adoption.

## Introduction

Evidence-based solutions and active involvement of all invested parties is recommended in optimal stroke care.^1^ Patient and public involvement (PPI) and/or engagement, often defined as research conducted “with” or “by” members of the public rather than “on,” “about,” or “for” them,^2–4^ actively engages patients, their families, and the public as co-researchers to make the research more relevant, credible, and impactful.^2,5–9^ While often confused with qualitative research or used interchangeably with co-design and participatory action research (where power is also redistributed between citizens and researchers), PPI partners are not study participants.^10^

Emergent PPI issues in research include inconsistent reporting, poor conceptualization, and limited information on context, processes, and impact provided.^11–13^ It has been contended that measuring PPI impact may not be possible and risks missing the intrinsic value of PPI in and of itself.^14,15^ However, existing evidence supports PPI as positively impacting research design and delivery and as being clinically impactful at the level of the individual in healthcare research. Scoping reviews in pre-clinical research, cancer, dementia, orthopedic, and occupational therapy populations identify limited formal evaluation of PPI and mostly anecdotal evidence supporting improved research relevance, research design, and research ethics.^16–21^ Standards for reporting PPI in research, for example, the Guidance for Reporting Involvement of Patients and the Public (GRIPP), and checklists have been developed to improve the quality of reported PPI and ensure consistency across the international literature.^8,22^

Stroke research has grown exponentially.^23^ For example, the number of studies indexed under stroke in PubMed in 2020 was five times greater than that in 2010.^24^ It is unknown whether adoption of PPI in stroke research has kept pace with this growth. Therefore, this scoping review aims to identify PPI uptake in empirical stroke research, summarize the available literature on how PPI is currently being implemented and reported, and identify any gaps. This scoping review actively involves a stroke survivor as a research partner, allowing their say in how this research is undertaken and providing unique insights.^25^

## Methods

### Design

As the extent and nature of the phenomenon under review remains largely unknown, a scoping review was conducted.^26^ Its purpose was to clarify the concept of PPI in the stroke literature; identify and map key characteristics or factors related to this concept and identify and analyze gaps in this emergent topic.^27^ This review followed published scoping review guidelines,^28–30^ adhering to the Preferred Reporting Items for Systematic Reviews and Meta-Analysis (PRISMA) extended guidelines and checklist for scoping reviews.^29,31^ The protocol was co-produced with a PPI partner and pre-published.^32^

### Identifying the research question

The overarching review question simply asks: What PPI activities are conducted and reported in stroke research? Included articles were interrogated with the following questions:

a) What types of stroke research utilize PPI?
b) How is PPI in stroke research reported?
c) What is the profile of PPI participants involved in stroke research, including representation of stroke-related disabilities?
d) Where in the research cycle is PPI being used?
e) What barriers and facilitators to PPI in stroke research are documented?
f) What impact from PPI activities is documented?

### Population, concept, and context (PCC) of the scoping review

The PCC structure helped define the review,^28^ as per the published protocol:^32^

a) Population: individuals with lived experience of stroke, including stroke survivors, carers, and family members.
b) Concept: PPI as described.^2,3^
c) Context: empirical stroke research.^1^

### Search strategy and inclusion/exclusion criteria

A comprehensive PCC search strategy was developed with a library liaison officer. Electronic databases, searched to January 2023, included PubMed, CINAHL, EMBASE, and PsycINFO. Gray literature databases (Leanus, OpenGrey, and relevant stroke organizational websites) were searched for additional relevant research. A sample search strategy is provided (Supplemental file 1).

Inclusion criteria comprised:

a) Studies published from 2014 onwards. This criterion was imposed based on the volume of papers returned during searches from database inception and the clear cutoff timepoint provided by the first PPI in research guideline publication;
b) Empirical stroke research including qualitative, quantitative, and mixed methods and, where directly referenced in the empirical studies, associated published consultation/PPI processes;
c) English language publications;
d) Studies documenting PPI activity/initiatives fitting the principles of PPI in research,^2,3^ irrespective of terminology.

Exclusion criteria:

a) Studies that did not explicitly involve PPI partners;
b) Studies reporting mixed populations or conditions other than stroke;
c) Publications that did produce original empirical data (e.g., editorials, commentary pieces) and conference abstracts;
d) Studies mistakenly reporting PPI activity: where the people with lived experience of stroke were study participants, not co-researchers.

### Screening process

Identified studies were collated (duplicates removed) in Covidence review software and screened independently by title and abstract by two reviewers, drawn from a bank of reviewers (OL, PCP, RG, AR, LMC, POB, DM, CF, CDF). A conservative approach was taken whereby if the study met the empirical stroke research criterion, but PPI was not mentioned in the abstract, it progressed to full-text review. Full-text screening was independently conducted by two reviewers. A publication date cutoff from 2014 onwards was imposed at this stage. Conflicts arising between reviewers were resolved through wider group discussions (OL, PCP, CF, CDF).

### Extracting and charting the data

A data extraction pro forma was developed for charting and extracting data,^28^ aligned to the research questions. Study design was considered across the continuum from descriptive to exploratory to explanatory research as defined by Portney (2020).^33^ PPI activity was staged according to the research cycle framework^3^ and reporting of PPI activity checked against GRIPP2 short-form checklist^11^ mandatory items. Two researchers (PCP and CF) independently extracted data items to ensure consistency. Per scoping review guidelines, study quality was not formally assessed.^28^

### Data synthesis

Extracted data were tabulated under predefined headings, providing a summary of the primary studies. Adherence to reporting guidelines was collated in tabular format. Narrative and graphical syntheses were next conducted, identifying the type and geographical representation of stroke research using PPI; the profile of PPI participants; where in the research cycle PPI was included;^3^ and barriers and facilitators to PPI and its impact in stroke research.^11^

## Results

**Figure 1.**
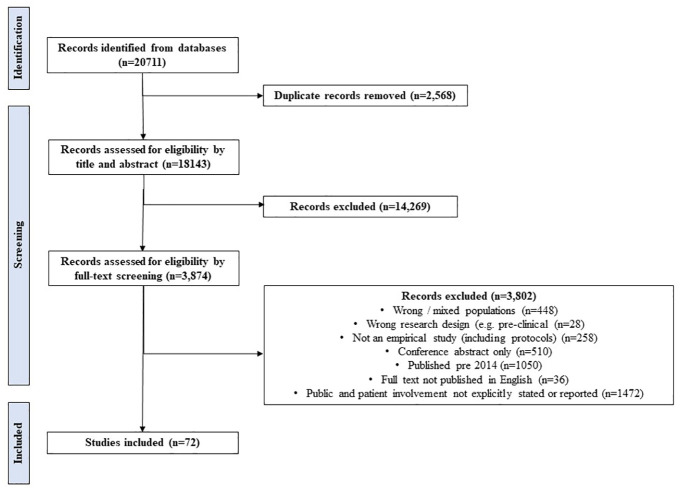
PRISMA flow chart depicting search results and study selection.

**Figure 2.**
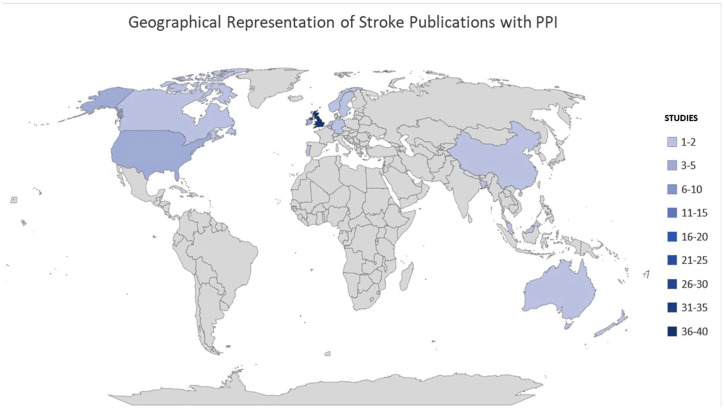
Global heatmap of publications with PPI in empirical stroke research.

Figure 1 details article retrieval, screening, and inclusion stages. From n = 18,143, unique studies identified across the databases from inception to January 2023, n = 3874, were eligible for full review (n = 2824 from 2014 onward). Only 2% (n = 72) of publications from 2014 onwards met the inclusion criteria.^34–105^ The included studies reporting PPI activity originated from high-income countries: United Kingdom^35,37,38,40,41,46–48,52,55–58,60,62,65,68,71,72,78–80,83,84,86,87,89,91–96,100–103^ (n = 37, 52%), Ireland^50,64,75–77^ (n = 5, 8%), United States^36,39,51,59,104^ (n = 5, 8%), Australia^44,74,90,99^ (n = 4, 6%), Canada^67,70,81^ (n = 3, 4%), Germany^43,63,85^ (n = 3, 4%), Sweden^53,61,66^ (n = 3, 4%), Norway^42,97^ (n = 2, 3%), Belgium^49^ (n = 1, 1%), Cyprus^45^ (n = 1, 1%), Netherlands^88^ (n = 1, 1%), Portugal^98^ (n = 1, 1%), and New Zealand^54^ (n = 1, 1%), higher-middle-income countries like China^69,73,105^ (n = 1, 1%) and Malaysia^34^ (n = 3, 4%), and one lower-middle-income country Bangladesh^82^ (n = 1, 1%; Figure 2).

A detailed table summarizing all included studies is available (Supplementary file 2), with a narrative summary addressing the review question provided below.

### Types of stroke research utilizing PPI

Descriptive research was most represented (qualitative n = 43; survey n = 10). Exploratory research designs identified included cohort (n = 4), methodological (n = 12), and correlational/predictive (n = 1) studies. Explanatory research, identified in six studies, described pragmatic clinical (n = 4), randomized control (n = 1), and quasi-experimental (n = 1) trials. Three studies synthesized existing data.

### PPI reporting in stroke research

Only 13% (n = 9) of manuscripts included a definition of their PPI concept. A greater percentage (67%; n = 48) detailed the PPI activities undertaken. The included studies’ compliance with the GRIPP2^11^ short-form checklist items for reporting PPI are detailed (Supplementary file 3). Five studies (7%) failed to report any items.^44,57,58,70,72^ One study (1%) cited the GRIPP2 checklist, eight studies (11%) demonstrated 100% compliance with all checklist items (mandating both positive and negative discussion and reflection items), and 25 studies (35%) partially reported all five checklist items. Across all studies, the aims (n = 65, 90%) and methods (n = 65, 90%) checklist items were most consistently reported. Notably, many studies (57%) failed to critically reflect on their PPI activities.

### Profile of PPI participants involved in stroke research

All studies provided information whereby PPI representation could be identified; 64% (n = 46) reported information about the profile of their PPI partners, primarily sex and/or age, with one study providing ethnicity. Where the number of PPI partners across the studies was reported, n = 285 were people with stroke (ages ranging from 32 to 91 years and time from stroke between 2 and 12 years); n = 44 were family members/carers; and n = 46 studies referenced public representatives/stroke support organizations. Stroke-related disability, where reported, reflected the research topic: n = 15 (21%) reported aphasia; n = 3 (4%) visual impairment; n = 3 (4%) cognitive difficulties; n = 1 (1%) mood issues; n = 1 (1%) motor weakness unspecified; and n = 1 (1%) upper-limb weakness. No study profiled wheelchair-use or mobility issues.

### PPI across the research cycle

**Figure 3.**
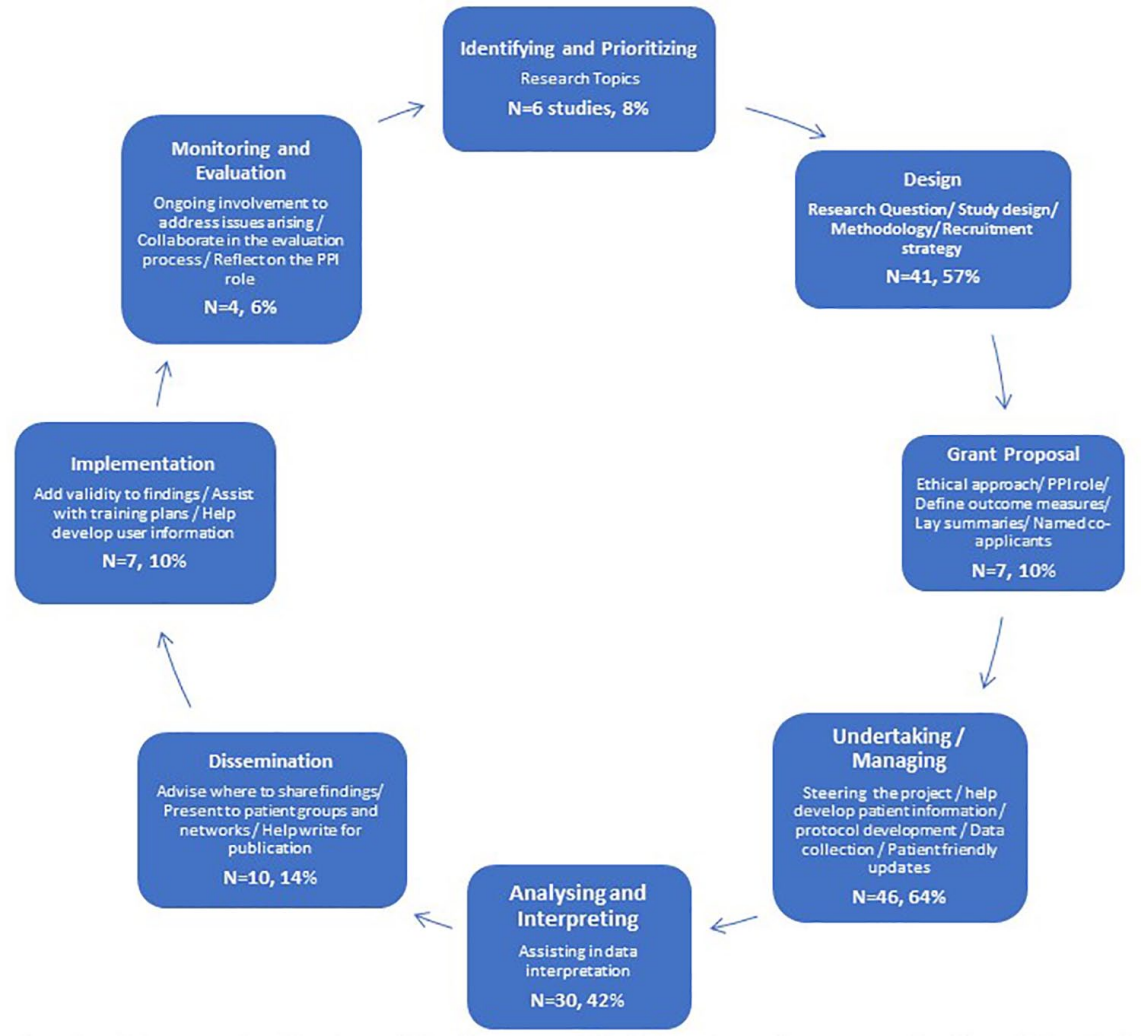
Frequency of reported PPI in empirical stroke across the research cycle stages.

Sixty-eight studies (94%) provided information about their PPI activities, summarized in Figure 3 under the published National Institute for Health and Care Research (NIHR) PPI research phases. Three studies (4%) integrated PPI across all research phases.^37,98,104^ The majority included PPI in undertaking/managing research (n = 46, 64%) and study design (n = 41, 57%). Five studies (7%) reported PPI contributions in the acknowledgment section or named a PPI co-author without elaboration,^44,57,58,70,72^ meaning activities/contributions to research cycle stages could not be identified.

### Documented barriers and facilitators to PPI in stroke research

**Figure 4.**
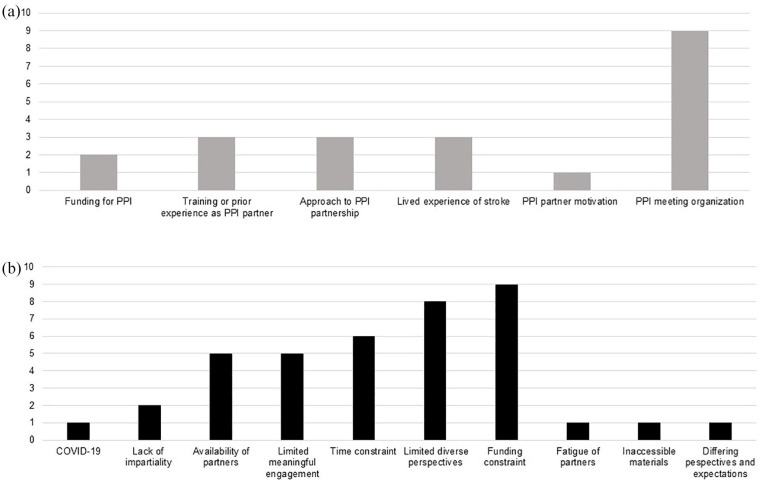
**(**a) Frequency of facilitators and (b) barriers to PPI reported in empirical stroke research.

Only 29% of studies (n = 21) reported facilitators and/or barriers to PPI integration in their research. The most frequently reported facilitator was well-organized PPI meetings, alongside aspects including trained/experienced PPI partners and authentic partnership (Figure 4(a)). Barriers to PPI in stroke research primarily reflected funding, limited PPI diversity, and time constraints (Figure 4(b)).

### Documented impacts of PPI activities

Of the studies (n = 40, 56%) reporting impact (Supplementary file 2), only positive items were documented including that the insights provided by PPI representatives improved the care of study participants, helped validate findings, kept patient perspectives central to the research process, and improved communication/lay-summaries of complex concepts.

## Discussion

This scoping review, conducted with its primary aim to clarify the concept of PPI in empirical stroke research,^27^ identifies poor uptake and reporting of PPI in stroke despite PPI being a well-supported research concept for over 20 years.^3,106–112^ Only 2% of articles reviewed reported PPI activity. The need for wider education in stroke research about PPI and facilitation of PPI activities in future stroke research is evident, alongside gaps identified in current PPI practices. PPI, where reported in stroke research, was feasible to embed in descriptive, exploratory, explanatory, and synthesis methods.

The United Kingdom dominates published PPI-embedded stroke research, as identified in previous scoping reviews addressing PPI.^17,18,113^ Results likely reflect the countries where policy-driven initiatives have promoted PPI in healthcare.^3,107–111^ All studies included in this review, with one exception, were conducted in developed countries. Results further emphasize the need for widespread support of PPI around the world, particularly in middle- and low-income countries, where 90% of all stroke-related deaths and the stroke burden (e.g. higher disability-adjusted life years)^114^ are concentrated.

Clinical trials alongside implementation science address efficacy and effectiveness of interventions. These make a substantial contribution to evidence-based care^33^ and are most likely to benefit from PPI.^115^ Only six clinical trials in stroke (8%) were included in this review, signaling a lack of PPI contribution to current high-level stroke research. Furthermore, four of these failed to sufficiently detail their PPI activities (see Supplemental Table 2). PPI in clinical trials can include research priority setting, study design (including selection of outcomes), as well as dissemination and implementation of trial findings.^116–118^ Evidence identifies PPI inclusion can improve recruitment and retention and increase the uptake of research findings by better meeting healthcare consumers’ needs.^116,119^ Person-centered care, characterized by a strong partnership between patient and clinician, is already strongly endorsed,^71^ but partnership is also needed in the scientific evidence that underpins care to be truly responsive to the needs and priorities of people with stroke.

Inconsistency was evident in how PPI was reported across studies. Often, quality of the engagement could not be determined (e.g. articles which included a PPI co-author or recognized PPI contribution in acknowledgements only), an approach that has been labeled as “virtue signaling” in the literature.^118^ This is important to address in future stroke research to eliminate current ambiguity on what constitutes PPI and its impact.^120^ Standardized reporting guidelines reduce time and reporting biases and increase manuscript quality and readers’ comprehension.^121^ We recommend PPI checklist items be adopted in stroke research reporting to ensure transparency and consistency across the international literature^8,22^ as inconsistencies were evident in this review’s findings. Indeed, authors of this review could not exclude studies by abstract, as PPI seldom appeared in this section.

Facilitators and barriers to PPI, with important considerations for future stroke research, were identified in n = 21 studies in this scoping review. Organization, particularly with respect to PPI meetings, was the most-cited facilitator to successful PPI-implementation, although capacity-building for PPI partners and adequate resourcing of PPI activities were deemed important. In contrast, funding constraints, time required, and limited representation from marginalized groups were commonly identified barriers. We observed a positive reporting bias for PPI in stroke research.^122^ One of two previously identified pitfalls, summarized as the tale of two towers, was identified: (1) The Tower of Babel, where medical jargon/paradigms propagate impressions of academic elitism (identified in this review as inaccessible PPI materials), and (2) The Ivory Tower (not identified), where critical inputs from outside academia that challenge views are unwelcome.^122^

Some limitations in this scoping review require consideration. We restricted our search to English language publications from 2014 onwards. It is possible we may have missed studies including PPI. We excluded articles that only described PPI methodologies and study protocols detailing PPI activity as they did not meet the criterion of empirical research. Where research cited a twinned article addressing its PPI methods, we did include this research. Many conference abstracts addressing PPI activities were eliminated. We justified these choices as consumers of research (clinicians, service funders/providers) usually seek empirical evidence, alongside patient preferences, to guide practice.^123–125^

In keeping with GRIPP2 reporting items, we briefly reflect on PPI in this scoping review. Our PPI partner and co-author provided insights that helped refine the published protocol and shape the review questions. The review findings, considered from the perspective of someone with lived experience of stroke and experience as an active PPI panel member, helped expand our discussion points. Our collaboration was facilitated by having a PPI partner with prior research experience and well-established working relations. PPI activities, conducted in personal time, required balancing work and family commitments.

## Conclusion and future research recommendations

There is poor uptake of PPI in published stroke research currently, notably in low- and middle-income countries. Where included, articles reporting PPI lack consistency in what they report and how they report it. To improve the quantity and quality of PPI-embedded empirical stroke research, several key recommendations are made:

- Senior stroke researchers must lead by example, welcoming PPI as a necessary cultural change in research conduct.
- Experiential knowledge of PPI partners should be considered in all research cycle phases to improve relevance, quality, and validity of stroke research and to ensure opportunity for equitable partnership and contribution as authors.
- Investment in training for researchers and PPI partners is required to build working relations, establish clear roles and responsibilities, and ensure transparency in communication and documentation.
- Financial remuneration that reflects PPI partners’ time is required.
- Ethical engagement with diverse stroke communities is needed to ensure adequate representation in PPI partners.
- PPI should be integral to ongoing global efforts to build stroke research capacity in low- and middle-income countries.
- PPI should become a standard item for journal submission, including a reporting checklist (e.g. GRIPP2) for quality and consistency, and PPI should be referenced in the abstract/keywords.

## Supplemental Material

sj-docx-1-wso-10.1177_17474930241262638 – Supplemental material for A scoping review of patient and public involvement in empirical stroke research Supplemental material, sj-docx-1-wso-10.1177_17474930241262638 for A scoping review of patient and public involvement in empirical stroke research by Paula da Cruz Peniche, Christina Danielli Coelho de Morais Faria, Patricia Hall, Caitriona Fingleton, Louise McPhillips, Rebecca Gaetz, Aaron Roche, Laura McCann, Padraig O’Beaglaoich, Diarmuid Murphy, Julianne Hickey and Olive Lennon in International Journal of Stroke

sj-docx-2-wso-10.1177_17474930241262638 – Supplemental material for A scoping review of patient and public involvement in empirical stroke research Supplemental material, sj-docx-2-wso-10.1177_17474930241262638 for A scoping review of patient and public involvement in empirical stroke research by Paula da Cruz Peniche, Christina Danielli Coelho de Morais Faria, Patricia Hall, Caitriona Fingleton, Louise McPhillips, Rebecca Gaetz, Aaron Roche, Laura McCann, Padraig O’Beaglaoich, Diarmuid Murphy, Julianne Hickey and Olive Lennon in International Journal of Stroke

sj-docx-3-wso-10.1177_17474930241262638 – Supplemental material for A scoping review of patient and public involvement in empirical stroke research Supplemental material, sj-docx-3-wso-10.1177_17474930241262638 for A scoping review of patient and public involvement in empirical stroke research by Paula da Cruz Peniche, Christina Danielli Coelho de Morais Faria, Patricia Hall, Caitriona Fingleton, Louise McPhillips, Rebecca Gaetz, Aaron Roche, Laura McCann, Padraig O’Beaglaoich, Diarmuid Murphy, Julianne Hickey and Olive Lennon in International Journal of Stroke
