# Supplementary material for: A scoping review of patient and public involvement in empirical stroke research
Source: Int J Stroke. 2024 Jul 31;19(9):962–72. doi: 10.1177/17474930241262638 (PMC11528947; doi:10.1177/17474930241262638)
Supplement: sj-docx-1-wso-10.1177_17474930241262638 – Supplemental material for A scoping review of patient and public involvement in empirical stroke research [file sj-docx-1-wso-10.1177_17474930241262638.docx]

Supplementary file 1: Example of search strategy

| **Population:**  **#3 = #1 AND #2** | #1: patient* OR survivor* OR adult* OR famil* OR carer* OR caregiver* OR parent* OR stepfamil* OR step-famil* OR young-adult* OR consumer |
| --- | --- |
|  | #2: stroke OR Poststroke OR "post-stroke" OR "Cerebrovascular disease" OR "Cerebrovascular disorder" OR CVD OR CVA OR “brain infarction” OR "Intracranial arterial diseases" OR TIA OR “transient ischemic attack” |
| **Concept: #4** | #4: “patient and public involvement” OR “patient involvement” OR “patient partnership” OR “patient collaboration” OR “patient engagement” OR “patient advocacy” OR “patient participation” OR “consumer participation” OR “consumer involvement” OR “consumer engagement” OR “stakeholder participation” OR “stakeholder engagement” OR “patient driven” OR “survivor participation” OR PPI OR PPIE OR "client participation" OR "experience based" OR "experience-based" OR experiential OR Consultation OR consultancy |
| **Context:**  **#7 = #5 AND #6** | #5: stroke OR Poststroke OR "post-stroke" OR "Cerebrovascular disease" OR "Cerebrovascular disorder" OR CVD OR CVA OR “brain infarction” OR "Intracranial arterial diseases" OR TIA OR “transient ischemic attack” |
|  | #6: research OR review OR investigate OR study OR project OR evaluation |
| **Final Search** | #3 AND #4 AND #7 |
