# Supplementary material for: A scoping review of patient and public involvement in empirical stroke research
Source: Int J Stroke. 2024 Jul 31;19(9):962–72. doi: 10.1177/17474930241262638 (PMC11528947; doi:10.1177/17474930241262638)
Supplement: sj-docx-2-wso-10.1177_17474930241262638 – Supplemental material for A scoping review of patient and public involvement in empirical stroke research [file sj-docx-2-wso-10.1177_17474930241262638.docx]

Supplemental file 2: **Table 2.** Description of included studies.

| **Citation; country** | **Study Objective** | **Research type** | **Public and Patient Involvement** | | |
| --- | --- | --- | --- | --- | --- |
|  |  |  | **1. PPI Concept reported (yes / no)**  **2. Approach** | **PPI partners:**  **1. Population**  **2. Profile** | **PPI valuation, benefits, and/or impact** |
| Ainuddin et al. 2021^34^  Malaysia | Synthesize evidence for rehabilitation in falls and risk of falls after stroke in Southeast Asia. | Synthesis of research (scoping review) and descriptive (qualitative) | **1.** No  **2.** Consultation by focus group | **1.** Patients (n=6)  Family/caregivers (n=7)  Public representative/support organization (n=7 healthcare professionals)  **2.** Public representative/support organization: 5 physiotherapists, 1 occupational therapist, and 1speech therapist; 1–5 years of professional experience. | - Scoping review was inadequate alone to gain a realistic view of practices in Southeast Asia. Stakeholder consultation provided added value.   . |
| Allen et al. 2019^35^  United Kingdom | Examine the utility of simulation and machine learning to enhance SSNAP clinical audit outputs. | Exploratory (methodological) | **1.** No  **2.** Meetings (3 times during the course of the project) | **1.** Patients or caregivers (n=5)  **2.** NR | NR |
| Andrews et al. 2018^36^  United States of America | Detail a novel approach to address the challenges of blending clinical care and research in a pragmatic clinical trial. | Descriptive (qualitative) | **1.** No  **2.** NR | **1.** Patients (n=2)  Family/caregivers (n=2)  **2.** NR | - Ensured consistency with Belmont principles and regulatory requirements in testing complex interventions. - Kept the patient perspective central throughout the process for the team and the institutional review board. - Helped ensure that patients received better care during the study; patients and their caregivers saw that healthcare systems continue to learn how to improve post-stroke care; and patients and caregivers’ choices were respected. |
| Aries et al. 2021^37^  United Kingdom | Describe the role of PPI in a mixed-methods feasibility study (MoTaStim-Foot). | Descriptive (qualitative) | **1.** Yes  **2.** A series of interactive workshops and regular meetings | **1.** Patients (n=9)*  Family/caregivers (n=9)*  Public representative/support organization (n=5)  *Numbers summed across workshops A and B  **2.** Patients: 3-12 years post-stroke  Public representative/support organization: 3 experienced clinicians and 2 academic members | - Authors identified standards for PPI that worked well /could be improved including implementation of appropriate support and learning, good communication, enabling PPI advisors to influence governance of the research and evaluating the impact of PPI. - PPI contributions were deemed essential to inform the funding application and important for study management and overall research process. - No specific negative effects with such intensive PPI levels. |
| Barnfield et al. 2017^38^  United Kingdom | Report the process of public involvement in the cocreation, improvement and dissemination of lay summaries in the COGFAST study. | Descriptive (qualitative and process evaluation) | **1.** Yes  **2.** Focus group | **1.** Patient (n=2)  Family/caregivers (n=3)  **2.** Patient: 1 female  Family/caregivers: 1 female | - Feasible and successful to prioritize and adapt lay summaries to communicate the findings of complex scientific research. - An evident strength was jargon identification missed by the academic team due to familiarity. - Insights likely to have resulted in more accessible resources for members of the public. - The authors endorsed the use of existing general guidelines on the production of lay summaries in conjunction with input from members of the public. |
| Bharmal et al. 2016^39^  United States of America | Describe the CPPR approach to increasing stroke awareness and elicit community perspectives serving minority communities about stroke prevention and research participation. | Descriptive (qualitative) | **1.** No  **2.** Meetings at least weekly by phone or in person | **1.** Public representative/support organization (n=16; 3 community partners, 1 staff, 2 students, 10 community members)  **2.** NR | - The majority of the members of the planning committee and Community Action Panel agreed knowledge about Los Angeles Stroke Intervention and Research Program increased and the symposium was valuable to their work. |
| Black et al. 2019^40^  United Kingdom | Examine the organization of centralized acute stroke at different times of day/week in services, and explore temporal consistency variations. | Descriptive (qualitative) | **1.** No  **2.** NR | **1.** Patient (n=2)  **2.** NR | NR |
| Blatchford et al. 2022^41^  United Kingdom | Understand the potential role of self-management programs such as My Life After Stroke. | Descriptive (survey and qualitative) | **1.** No  **2.** Consultation meetings | **1.** Patients (n=NR)  Family/caregivers (n=NR)  Public representative/support organization (n=NR)  **2.** Patients or family/caregivers: survivor and carer support groups in the community  Public representative/support organization: lay membership of the Programme Steering Committee and Intervention Development Group, a lay representative coapplicant, active engagement with The Stroke Association. | NR |
| Bogstrand et al. 2022^42^  Norway | Investigate multidisciplinary health professionals’ experiences of long-term services for patients with mild to moderate poststroke cognitive impairment in a North Norwegian context. | Descriptive (qualitative) | **1.** No  **2.** Discussion | **1.** Patients (n=NR)  Family/caregivers (n=NR)  Public representative/support organization (n=NR)  **2.** Patients or family/caregivers: from a stroke organization | NR |
| Busetto et al. 2022^43^  Germany | Identify the patient pathways in acute stroke, including interactions from the perspective of stroke patients. | Descriptive (qualitative) | **1.** No  **2.** Discussion | **1.** Patients (n=NR)  **2.** Patients: Patient Council of the Department of Neurology  **2.** NR | NR |
| Cadilhac et al. 2016^44^  Australia | Assess the effects of cognitive, language, or global impairments on participation in a stroke self-management program. | Exploratory (cohort) | **1.** No  **2.** NR | **1.** Advisory committee chair came from the National Stroke Foundation  **2.** NR. | NR |
| Charalambous et al. 2022^45^  Cyprus | Analyze and compare the views of and barriers and facilitators to PPI for people with aphasia and stroke survivors without aphasia. | Descriptive (qualitative) | **1.** Yes  **2.** Meetings | **1.** Patients (n=1)  **2.** Patients: 32-year-old female, with mild-moderate anomic aphasia; 5 years post stroke; master’s degree in Research Methodologies and Stroke Ambassador of the Cyprus Stroke Association; the community social worker under the auspices of the European program “Solidary Network in Action” for the Municipality of Nicosia (Cyprus). | - The PPI partner helped the study team formulate the research questions and actively contributed to the study’s methodology. - She reported a positive PPI experience during the study as co-researcher and felt well-supported. - She was a key player in the consensus meeting for the emergence of the four themes. |
| Clarke et al.  2021^46^  United Kingdom | Report the process evaluation of the Collaborative Rehabilitation in Acute Stroke (CREATE) Study. | Descriptive (qualitative) | **1.** No  **2.** NR | **1.** Patient (n=NR)  Family/caregivers (n=NR)  **2.** NR | - Provided important insights about participants’ involvement that would not have been known otherwise |
| Donetto et al. 2021^47^  United Kingdom | Discuss the liminality lens in understanding and interpreting behaviors and interactions between co-design participants for space and place in the stroke unit. | Descriptive (qualitative) | **1.** No  **2.** Participatory approach using  interviews, events, and co-design groups | **1.** Patients (n=14)  Family/caregivers (n=9)  Public representative/support organization (n=21)  Numbers summed across EBCD cycles  **2.** NR | - Visible changes to the environment were implemented as product of collective action including family members - Codesign and partnership continued beyond the celebration event. |
| Eltringham et al. 2019^48^  United Kingdom | Explore and include the experiences of people with swallowing difficulties following stroke and informal caregivers in understanding delivery of care. | Descriptive (qualitative) | **1.** Yes  **2.** Participatory methods approach | **1.** Public representative/support organization (n=3)  **2.** NR | - Enriched the themes identified by the researcher, allowed validity checking of the conclusions from a stroke survivors perspective and highlighted findings that were relevant to people a affected by stroke. - Identified the frequency of feeling words used by the participants describing their reactions to the stroke event and how they felt about the medical interventions and care processes. - Empathized with findings detailing lack of communication to patients about the importance of adequate nutrition and hydration and they perceived a lack of standardization in procedures and communication about the risk of pneumonia and transitioning from short term to long term nutrition. - Future improvements: sometimes members brought up things from their own experiences, which had not always been raised by the interview participants; and learning outcome for the future would be to include more time for building research skills and discussion |
| Espinoza et al. 2016^49^  Belgium | **1.** Describe the practices and experiences with development and implementation of the Prehospital Stroke Study (PreSSUB) of in-ambulance telestroke;  **2.** Report PreSSUB-I pilot study results. | Exploratory (methodological) | **1.** No  **2.** Interview | **1.** Patients (n=NR)  Public representative/support organization (n=NR)  **2.** NR | NR |
| Franklin et al. 2017^50^  Ireland | Identify the top 10 research priorities for long-term aphasia. | Descriptive (survey and qualitative) | **1.** Yes  **2.** Survey and consensus meetings | **1.** Patients (n=31)*  Family/caregivers (n=10)*  Public representative/support organization (n=27)  *Numbers summed across survey and consensus  **2.** Patients: 4 mild, 11 moderate, and 6 severe aphasia; among the other 10 patients, at least one individual with Wernicke-type aphasia, ages ranged from 48-91 years.  Public representative/support organization: 27 speech and language therapists (hospital and community therapists) | - Top 10 research priorities relating to aphasia were identified by key stakeholders. - Demonstrated that people with aphasia are fully able to participate in research priority setting. - Using this methodology, rather than just “be researched”, people with aphasia were able to add their own knowledge, experience and perspective to “what should be researched”. The authors believe this is an important first step to ensuring that future aphasia research reflects what is important to those affected |
| Gesell et al. 2020^51^  United States of America | Describe stakeholders’ contributions to the design, conduct, and dissemination of findings from a multicenter pragmatic clinical trial. | Descriptive (qualitative) | **1.** No  **2.** Focus groups and group discussions | **1.** Patients (n=NR)  Family/caregivers (n=NR)  Public representative/support organization (n=NR)  **2.** Patients: female, African American, and rural North Carolinians.  Family/caregivers: represented varied income and educational levels and rural/urban areas.  Public representative/support organization: hospitals, health systems, and training institutions: all relevant members of hospital based stroke teams (rural/urban areas), quality/performance improvement coordinators, case managers/social workers, nurse managers, patient education directors and senior hospital administrators. Community-based services: networks were aging service providers, caregiver support services providers and pharmacists (rural/urban areas). Advocacy organization, and policymakers: influential leaders involved in high level advising to support dissemination and sustainability (examples: National advocacy organization and Regional networks of health and  human service providers | - Engagement resulted in important changes to aspects of post-acute stroke care represented by stakeholder’s perspectives. - The diverse leadership team provided perspectives that were often different and sometimes even opposing, deemed to have resulted in better problem solving and products. - Multiple study points where stakeholders drove engagement activities that clearly strengthened the study. - Inclusion of stakeholders in the design and implementation of future studies and programs recommended. |
| Golding-Day et al. 2022^52^  United Kingdom | Develop an online questionnaire and conduct a national survey to collate the views of orthotists, physiotherapists, and occupational therapists, on current practice and their perceptions of factors determining the delivery of orthotic interventions to stroke patients in the United Kingdom. | Exploratory (methodological) and descriptive (survey) | **1.** No  **2.** NR | **1.** Public representative/support organization (n=NR)  **2.** Public representative/support organization: a team of stroke clinicians including an orthotist, physiotherapist and occupational therapists, and stroke clinical academic researchers, and members of the Nottingham Stroke Research Partnership Group. | - The public representative (an orthotist and two occupational therapists) and stroke partnership group influenced the research team by their clinical and lived experiences of orthotic interventions. |
| Gustavsson et al. 2020^53^  Sweden | To explore healthcare professionals’ use / potential use of ICT to enable person-centred stroke rehabilitation. | Descriptive (qualitative) | **1.** No  **2.** NR | **1.** Public representative/support organization (n=1)  **2.** NR | NR |
| Hale et al. 2014^54^  New  Zealand | Adaptation of the Bridges stroke self-management program for people with stroke in New Zealand, and exploration of its acceptability to patients, extended family (whānau), health professionals, and support services. | Descriptive (qualitative) | **1.** No  **2.** Consultation and focus group | **1.** Patients (n=60)*  Family/caregivers (n=NR)  Public representative/support organization (n=17)*  *reported in study for one phase only  **2.** Patients: age 50-80 years  Family/caregivers: NR  Public representative/support organization: managers and field officers of local branches and the central office of  the Stroke Foundation of New Zealand in six towns and cities, physiotherapists and occupational therapists working in neurorehabilitation and the Stroke Foundation of New Zealand in three cities and towns | NR |
| Hazelton et al., 2022^55^  United Kingdom | Assess the effectiveness of interventions addressing perceptual disorders after stroke on measures of performance in activities of daily living when compared to no intervention or control | Synthesis of research (systematic review) | **1.** No  **2.** 'Top and tail' approach | **1.** Patients (n=5)  Public representative/support organization (n=4)  **2.** Patients: people with lived experience of stroke  Public representative/support organization: expert clinical knowledge | - Following a detailed discussion with the stakeholder group, the authors refined and clarified the scope of the definition to include disorders affecting six areas. - In discussion with the stakeholder group, the authors decided how to address more complex, stroke-related disorders. |
| Hepworth et al. 2019^56^  United Kingdom | Develop a patient reported vision-related quality of life outcome measure after stroke. | Exploratory (methodological) | **1.** No  **2.** Consultation, nominal group session and Delphi approach | **1.** Patients (n=177)*  Public representative/support organization (n=64)*  *summed across study phases  **2.** Patients: stroke survivors with visual impairment  Public representative/support organization: eye specialists involved in stroke care (orthoptists, ophthalmologist, and occupational therapist) | NR |
| Hepworth et al. 2021^57^  United Kingdom | Explore the reported symptoms of post-stroke visual impairment using epidemiology data. | Exploratory (cohort) | **1.** No  **2.** NR | **1.** NR (PPI coauthor named only)  **2.** NR | NR |
| Heron et al. 2017^58^  United Kingdom | Assess the feasibility of a novel adapted home-based cardiac rehabilitation program. | Explanatory (pragmatic clinical trial) | **1.** No  **2.** NR | **1.** NR (PPI coauthor named only)  **2.** NR | NR |
| Hinckley et al. 2014^59^  United States of America | Incorporate the views of people with aphasia on current research needs. | Descriptive (qualitative) | **1.** Yes  **2.** Nominal group technique | **1.** Patients (n=25 Suncoast Aphasia Support Group Members)  **2.** Patients: between year and 16 years post onset, with a range of types of severities of aphasia. | - Topics of importance to researchers that were not of particular importance to those living with impairment were identified underscoring the need for joint research priority areas. - Better consideration for dissemination of best practices and aphasia research results in an accessible manner for individuals with aphasia. - Productive process that enabled meaningful contribution to the discussion, review and edit of results. |
| Howard et al. 2021^60^  United Kingdom | Determine factors predicting adaptation to visual impairment after stroke from an early stage. | Exploratory (cohort) | **1.** No  **2.** Consultation and regular involvement | **1.** Patients (n=NR)  Public representative/support organization (at least 5)  **2.** Patients: Stroke survivors with visual impairment  Public representative/support: members of VISable organization | NR |
| Hu et al. 2022^61^  Sweden | Develop a digital tool, Rehabkompassen® and conduct preliminary evaluation of the usability and feasibility of the developed instrument for both stroke patients and health care professionals in an out-patient clinical setting. | Exploratory (methodological) an descriptive (qualitative) | **1.** No  **2.** Iterative and participatory design process | **1.** Patients (n=NR)  Public representative/support organization (n=NR)  **2.** Patients: NR  Public representative/support organization: multi-professional medical staff with expertise in stroke rehabilitation, experts in biomedical engineering and human interaction. | - Based on the feedback from both patients and physicians, the instrument developed allowed rapid capture of a patient’s unmet rehabilitation needs in clinical practice. - Both patients and medical staff involved in the development and evaluation of the instrument provided extra strength to the study. |
| Johnson et al. 2022^62^  United Kingdom | Develop an evidence-based, theory-driven, structured self-management programme for stroke survivors to address their unmet psychosocial and information needs about stroke and stroke management. | Descriptive (qualitative) | **1.** No  **2.** Consultation workshops | **1.** Patients*  Family/caregivers*  Public representative/support organization (n=12)  *n=4-8 in total  **2.** Patients: NR  Family/caregivers: NR  Public representative/support organization: healthcare professionals (2 physiotherapists, 2 nurses, 1 occupational therapist, 1 speech and language therapist, 1 support worker and 2 consultants), Stroke Association manager, Stroke Association group leader and the Early Supported Discharge Stroke manager. | - Consultation highlighted priority areas for the self-management programme. |
| Kampling et al. 2020^63^  Germany | Describe the processes for development of practice guidelines to support decision-making by a multidisciplinary team in stroke rehabilitation in individual cases. | Descriptive (survey and qualitative) | **1.** No  **2.** Workshop and focus group | **1.** Patients (n=5)*  Summed across phases where reported; NR for focus group study.  Public representative/support organization (n=18)  **2.** Patients: patients who had a stroke with mild to moderate cognitive impairment.  Public representative/support organization: experts with expertise in psychology, neurorehabilitation and rehabilitation, and/or neurosciences (3 clinical psychologists, 3 psychologists specialized in psychological (and occupational) research, 3 physicians, and 1 therapist for occupational, speech and physical therapy specialized in stroke care, respectively as well as a nurse representative, and an expert on rehabilitation) and the head psychologist of the German Statutory Pension. | NR |
| Kearns et al. 2019^64^  Ireland | **1.** Co-develop a feedback questionnaire for people with aphasia who undertake ICT-delivered aphasia rehabilitation.  **2.** Describe the collaborative co-design process.  **3.** Explore the experiences of the co-designers in the collaborative design process. | Exploratory (methodological) and descriptive (qualitative) | **1.** Yes  **2.** Collaborative co-design process through workshops | **1.** Patients (n=6)  **2.** Patients: 5 males, ages ranged from 43 to 76 years (60.7±11.3 years), 1.9–11.4 years post-stroke (5.8±3.8 years), presenting with mild to severe aphasia. | - Co-design, grounded on collaboration between those with a lived experience of aphasia and an SLT facilitator with expertise in ICT-delivered aphasia rehabilitation, resulted in a final product that reflects this collaboration. - Co-design provided opportunities for social interaction with other people with aphasia and allowed co-designers to reflect on their own abilities. - The design process, including the interactions, was engaging and contributions were valued. - The development of a feedback questionnaire was time and labor intensive but was an appropriate and feasible approach that was valued by all involved. - The process was inclusive, co-designers reported feeling comfortable about contributing in the workshops, reflected in individual exit interviews. - People with aphasia can, and should, be included in all stages of aphasia research processes. |
| King et al. 2020^65^  United Kingdom | Project future incidence, prevalence and cost of stroke among people aged 45 and over in the United Kingdom. | Exploratory /Observational (correlational /predictive) | **1.** No  **2.** Consultation through Delphi approach | **1.** Patients (n=NR)  Public representative/support organization (n=2)  **2.** Public representative/support organization: members of Stroke Association | NR |
| Kjörk et al. 2022^66^  Sweden | Develop a digital previsit tool and explore potential end user’s perceptions prior to testing it in a clinical setting, using a participatory approach with stroke as a case example. | Exploratory (methodological) | **1.** No  **2.** Participatory co-design approach | **1.** Patients (n=1)  **2.** NR | - The participatory approach, using service design principles, led to the creation of a tool based on user needs. |
| Kwok et al., 2023^67^  Canada | Explore patient and therapist perspectives of outcome measures used post-stroke. | Descriptive (qualitative) | **1.** No  **2.** NR | **1.** Patients (n=3)  **2.** Patients: experience of a severe stroke, ranging from two to ten years prior to the start of the research, with a variety of stroke consequences, including motor weakness and aphasia. | - This research significantly benefitted from the addition of three patient partners, who ensured the work reflected the patient experience. |
| Kyle et al. 2020^68^  United Kingdom | Examine stroke care managers and health professionals’ barriers and enablers to minimizing treatment burden and maximizing patient capacity. | Descriptive (qualitative) | **1.** No  **2.** Interviews | **1.** Patients  Family/caregivers  Public representative/support organization  n=4 in total  **2.** NR | NR |
| Lam et al. 2022^69^  China | Understand stroke survivors’ expectations and perceptions of a home-based virtual clinic, and (ii) survivors’ perceived facilitators of and barriers to receiving care at this virtual clinic. | Descriptive (qualitative) | **1.** No  **2.** NR | **1.** Patients (n=2)  **2.** NR | NR |
| Langstaff et al. 2014^70^  Canada | To examine hospital length of stay and readmission rates following improved access to timely intensive home-based stroke rehabilitation. | Explanatory (quasi-experimental) | **1.** No  **2.** NR | **1.** NR specifically  **2.** Members of the Regional Stroke Steering Committee and partners of the Stroke Network of Southeastern Ontario. | NR |
| Lawrence et al. 2019^71^  United Kingdom | Generate an evidence-based definition of non-pharmacological/non-surgical stroke secondary prevention. | Exploratory (methodological) | **1.** No  **2.** A structured, iterative process through Delphi approach | **1.** Public representative/support organization (n=NR)  **2.** NR | Consultation enabled Delphi participants to consider the relevance and meaningfulness of agreed key elements and draft definitions from stakeholders’ perspectives. |
| Lievesley et al. 2022^72^  United Kingdom | Describe the methods and outputs of a multidisciplinary research team who developed an oral health intervention. | Descriptive (qualitative) | **1.** No  **2.** NR | **1.** NR (PPI coauthor named only)  **2.** NR | NR |
| Luo et al. 2015^73^  China | Develop a PROM for patients with stroke that captures the patient’s viewpoint. | Exploratory (methodological) | **1.** No  **2.** In-depth open-ended interviews | **1.** Patients (n=20)*  Public representative/support organization (n=3)  *Summed across study phases  **2.** Patients**: 8 female, 3 with age ≤45 years, 7 with ages between 45-65 years, and 5 with ages ≥65, with varying educational levels  **Profile absent for one study phase | Identifying the items of the Stroke-PROM by including face-to-face interviews with patients strengthened the content validity of the preliminary scale. |
| Lynch et al. 2021^74^  Australia | Describe the process incorporating consumers and health professionals to systematically prioritize guideline recommendations for implementation. | Descriptive (survey) | **1.** No  **2.** Consultation through workshops and surveys | **1.** Patients (n=5)  Family/caregivers (n=1)  Public representative/support organization (n=104)  Unspecified consumers (n=18)  **2.** Patients: NR  Family/caregivers: NR  Public representative/support organization: 81% female, median age 36  Consumers: 80% female, median age 51 | - Feasible to implement an inclusive process, including patients and caregivers to systematically prioritize guideline recommendations for implementation. - Helps reveal a collective vision, overcome barriers and facilitate collective action towards successful implementation. |
| Manning et al. 2021^75^  Ireland | Address under-representation of the perspectives of working aged adults with post-stroke aphasia in social participation and living well with aphasia. | Descriptive (qualitative) | **1.** No  **2.** NR | **1.** Patients (n=4)  **2.** Patients: 3 females, working age living with post-stroke aphasia for 3–30 years | - Improved the validity and transferability of the findings, with relevance for adults with post-stroke aphasia in other countries, and other patient groups with communication impairment from other etiologies. |
| Manning et al. 2022a^76^  Ireland | Explore the perspectives of adults with post-stroke aphasia on what has or would help them to live well. | Descriptive (qualitative) | **1.** No  **2.** Participatory research approach | **1.** Patients (n=4)  **2.** Patients: 3 females, working age living with post-stroke aphasia for 3–30 years | - Accessible, inclusive interviews were designed to facilitate meaningful participation of adults with post-stroke aphasia, including those with severe aphasia, and to obtain rich, relevant information. |
| Manning et al. 2022b^77^  Ireland | Explore the ways in which people emotionally integrate and regulate negative psychological responses to aphasia. | Descriptive (qualitative) | **1.** No  **2.** Participatory learning and action tools | **1.** Patients (n=4)  **2.** Patients: Individuals with post-stroke aphasia | NR |
| Marshall et al. 2020^78^  United Kingdom | Trial of EVA Park (delivery platform) hosting a remote support group intervention. | Explanatory (pragmatic clinical trial) | **1.** No  **2.** NR | **1.** Patients (n=4)  Family/caregivers (n=1)  Public representative/support organization (n=3)  **2.** Patients: with aphasia | NR |
| McCormick et al. 2022^79^  United Kingdom | Report the development of the See, Imagine, Move: Upper Limb Action Therapy (SIMULATe) iPadTM- based application and the results of an early feasibility study. | Explanatory (pragmatic clinical trial) | **1.** No  **2.** Steering group meetings | **1.** Patients (n=NR)*  Family/caregivers (n=NR)*  Public representative/support organization (n=2)  *public and patient involvement and engagement (PPIE) representatives (n=3)  **2.** Public representative/support organization: a Stroke Association UK Coordinator, and an occupational  therapist | - The findings suggest highly usable technology-dependent therapy can be designed and developed with people living with stroke, for people living with stroke. |
| McKevitt et al. 2015^80^  United Kingdom | Investigate stroke survivors’ research awareness, use of research evidence in their medical care and willingness to be involved in research processes. | Descriptive (survey) | **1.** No  **2.** Discussions | **1.** Patients (n=24)  **2.** Patients: members of an existing stroke patient research group | - An appropriate strategy was used to design and test the questionnaire by working with members of the target population. |
| Mir et al. 2018^81^  Canada | Synthesize  the evidence supporting PFO closure plus antiplatelet therapy, antiplatelet therapy alone and anticoagulation alone on patients aged <60 years with cryptogenic stroke and PFO. | Synthesis of research (systematic review) | **1.** No  **2.** NR | **1.** Patients (n=3)  Public representative/support organization (n=NR)  **2.** Patients: had experienced a cryptogenic stroke (two of whom had undergone PFO closure)  Public representative/support organization: BMJ Rapid Recommendation panel | The patients stressed the importance of several outcomes and uniquely highlighted the importance of detailed information about the device or procedure-related adverse events. |
| Mondal et al. 2022^82^  Bangladesh | Gather evidence on the current prevalence and risk factors of stroke among Bangladeshi population using a survey instrument informed by people with stroke. | Descriptive  (survey) | **1.** No  **2.** Discussion | **1.** Patients (n=NR)  Family/caregivers (n= NR)  **2.** NR | NR |
| Morris et al. 2016^83^  United Kingdom | Explore how community stroke staff understand their role and respond to the psychological needs of stroke patients and carers. | Descriptive (qualitative and survey) | **1.** No  **2.** Focus group | **1.** Public representative/support organization (n=22)  **2.** Public representative/support organization: psychologists, 3 Stroke Association co-ordinators and two volunteers, 23 females, average age 40.0 or average age 46.8 or average age 45.7 years; average length of service 8.2 or 3.2 years or 8.4 years; 4 of the staff were in management roles, the remainder had roles described as “stroke co-ordinators”, “peer support co-ordinators”, “communications support coordinators”. 7 members of the groups had current membership of professional bodies. 14 had qualifications in health and social care ranging from professional nursing and social work qualifications to certificates in specific therapies. 3 held management qualifications. | - Participant consultation to frame questions ensured that lack of familiarity with particular technical terms and techniques was not an impediment and that the language used was appropriate for the population. - However, this did entail the avoidance of some common terms and reference to specific intervention and assessment procedures. |
| Morris et al. 2022^84^  United Kingdom | Describe initial development of We Walk, a multistage project to develop and pilot a theory-based, person-centred, dyadic behaviour change intervention to promote PA through outdoor walking in community-dwelling people with stroke. | Descriptive (qualitative) | **1.** No  **2.** NR | **1.** Patients (n=4)  Family/caregivers (n=NR)  Public representative/support organization (n=NR)  **2.** Patients: males with stroke  Family/caregivers: patients’ female companions  Public representative/support organization: members of two Scottish Chest Heart and Stroke Scotland support groups and local exercise groups. | - PPI helped inform final intervention refinement prior to piloting. |
| Nave et al. 2019^85^  Germany | Determine the efficacy of aerobic treadmill based, physical fitness training on maximal walking speed and activities of daily living compared to relaxation. | Explanatory (randomized controlled trial) | **1.** No  **2.** NR | **1.** Patient (n=1)  **2.** NR | NR |
| Patchick et al. 2015^86^  United Kingdom | Inform the development of a PROM for cognition by exploring stroke-survivor perspectives on the important, measurable impacts of persisting post-stroke cognitive problems. | Descriptive (qualitative) | **1.** No  **2.** Consultation | **1.** Patients (n=NR)  **2.** Patients: who had experience cognitive problems | - Communication aids were developed because of PPI input and guidance made qualitative interviews with cognitively impaired stroke survivors less challenging and possible. - PPI influenced the decision to interview stroke survivors independently of their carers as service users felt that a more open and honest dialogue would be achieved one-to-one. - The interview schedule was refined through pilot testing with cognitively impaired service users as part of the PPI process. This had the added benefit of providing training for researchers. |
| Porat et al.  2019^87^  United Kingdom | Identify stakeholders’ priorities and information needs in long-term stroke care. Codesign and evaluate an intervention integrated with the electronic health record system. | Descriptive (qualitative) | **1.** No  **2.** Active feedback using focus groups, face-to-face interviews and  usability evaluations | **1.** Patients or family/caregivers (n=19)  Public representative/support organization (n=30)  **2.** Patients: 4 female, average age of 65.5±11.4 years, ranging between 49–81; all had hypertension, 2 had heart problems, 1 was suffering from depression, 4 had mobility issues and 4 had minor cognitive deficiencies (attention and memory))  Public representative/support organization: 16 health and social care professionals, 6 commissioners and policymakers, and 8 general practitioners. General practitioners (4 men, 4 female, average of 10.3 years of experience in providing care to stroke survivors). | Engaging stakeholders throughout the design and evaluation process ensured that the intervention (features and functions) was in line with the needs reported by stroke survivors. |
| Prick et al., 2022^88^  Netherlands | Develop a patient decision aid with integrated outcome information for hospitalized patients with stroke. | Exploratory (methodological) | **1.** No  **2.** Co‑creation sessions | **1.** Patients (n=1)  Public representative/support organization (n=NR)  **2.** Patients: NR  Public representative/support organization: neurologists, rehabilitation specialists, geriatricians, a stroke nurse, an occupational therapist and a speech therapist | NR |
| Rai et al. 2021^89^  United Kingdom | Optimization of a digital intervention for stroke patients a person-based approach with PPI. | Descriptive (qualitative) | **1.** Yes  **2.** NR | **1.** Patients (n=2)  Public representative/support organization (n=2; Different Strokes Southampton and the Oxford Aphasia Group)  **2.** Public representative/support organization: . a senior representative of Speakeasy (a charity supporting people with aphasia) | - Essential contributions to the design of the intervention and research decisions from an early stage. - Drove a person-based approach that will hopefully inform wider discussions regarding the development of complex health interventions to make them attractive, engaging, useable and most importantly beneficial, for the widest range of people. |
| Ramage et al. 2022^90^  Australia | **1.** Provide practical guidance for researchers to co-produce interventions ready for clinical trial  **2.** Describe, as an exemplar, the development of the intervention package. | Descriptive (qualitative) | **1.** NR  **2.** Co-production | **1.** Patients (n=1)  Public representative/support organization (n=4)  **2.** Patients: NR  **2.** Public representative/support organization: clinicians with research training and experience | - Early involvement of knowledge users within the co-production team was deemed critical in guiding the development of a more broadly acceptable intervention. - The authors felt the key elements, adaptations, or refinements resulting from knowledge user input played a crucial role in the success of the intervention. - The authors felt the process was a worthy investment of resources. |
| Rowe et al. 2017^91^  United Kingdom | Survey orthoptists’ practice internationally in care provision for poststroke visual impairment. | Descriptive (survey) | **1.** No  **2.** Consultation | **1.** Public representative/support organization (n=NR; identified through non-specified stroke user forums in the United Kingdom)  **2.** NR | NR |
| Rowe et al. 2022^92^  United Kingdom | Co-develop a stroke-vision care pathway  for stroke survivors with visual impairment. | Descriptive (qualitative) | **1.** No  **2.** NR | **1.** Patients (n=3)  Public representative/support organization (n=5)  **2.** Public representative/support organization: 3 research and clinically active orthoptists, 1 neuro-ophthalmologist, 1 orthoptic professional society representative | NR |
| Sadler et al. 2016^93^  United Kingdom | Development and preliminary evaluation of a novel intervention to promote resilience after stroke. | Descriptive (qualitative) and explanatory (pragmatic clinical trial) | **1.** No  **2.** Consultation and group discussion | **1.** Patients (n=NR)  Public representative/support organization (n=NR)  **2.** Public representative/support organization: service user representatives from the King’s College London Stroke Research Patients and Family Group | - Engaging service users in co-designing appropriate interventions improved their relevance and impact for longer-term outcomes in stroke and other populations. |
| Sadler et al. 2017^94^  United Kingdom | Develop a process to engage stakeholders in the use of clinical and research data to co-produce solutions to improve long-term care for stroke survivors with multimorbidity. | Descriptive (qualitative) | **1.** No  **2.** Group meetings, focus groups, nominal group techniques and interviews | **1.** Patients  Family/caregivers  Public representative/support organization  Total n=37  **2.** NR | - The stakeholder engagement study adds to the co-production literature in terms of reflecting on the benefits and methodological challenges of undertaking research informed by a co-production approach to improve health care, which has received little attention. - The process developed potential solutions and pragmatically prioritized those solutions in collaboration with users and providers of stroke services. Further work is required to evaluate the impact and implementation of co-produced data-driven interventions for long-term stroke survivors. |
| Shiggins et al., 2022^95^  United Kingdom | Produce a Patient Research Experience Survey that would be accessible to people with aphasia. | Descriptive (qualitative) | **1.** Yes  **2.** Co-production | **1.** Patients (n=NR)  Family/caregivers (n=NR)  Public representative/support organization (n=NR)  **2.** Patients: people with aphasia with varying presentations and severities of aphasia (mild-severe)  Family/caregivers: NR  Public representative/support organization: academics, a clinician, and a representative from the NIHR | - The project provides an example of how the research feedback mechanism can be made more accessible and inclusive, while working collaboratively with a range of stakeholders. |
| Smith et al. 2018^96^  United Kingdom | Investigate the experiences and ongoing needs of community‐dwelling stroke survivors with upper limb dysfunction and that of their caregivers. | Descriptive (qualitative) | **1.** No  **2.** NR | **1.** Patients (n=NR)  Family/caregivers (n=NR)  **2.** NR | NR |
| Solbakken et al., 2022^97^  Norway | Describe the pragmatic priority setting process used to identify a prioritized top 10 list of research needs around communication and collaboration in transitional care for patients with acute stroke (TracStroke). | Descriptive (qualitative) | **1.** No  **2.** NR | **1.** Public representative/support organization (n=NR)  **2.** Public representative/support organization: health personnel and organizations for persons with stroke | NR |
| Sousa et al. 2019^98^  Portugal | Collate national data on access to and delivery rates for acute stroke unit care, intravenous thrombolysis and endovascular treatment throughout Europe. | Descriptive (survey) | **1.** No  **2.** NR | **1.** Public representative/support organization (n=NR)  **2.** Public representative/support organization: Representative from SAFE patient organization | NR |
| Thayabaranathan et al. 2022^99^  Australia | Describe the processes involved in co-designing a contextually appropriate yoga-based MBI for survivors of stroke and its formative evaluation. | Descriptive (qualitative) | **1.** No  **2.** NR | **1.** Patients (n=3)  Family/caregivers (n=1)  Public representative/support organization (n=NR)  **2.** Patients: NR  Family/caregivers: NR  Public representative/support organization: a clinical neuropsychologist, epidemiologist, statistician, exercise physiologist, occupational therapists, physiotherapists, yoga experts, basic scientists, and a neuroscientist | - The advisory committee helped ensure the manual-based program would be practical, achievable, and inclusive for participants of varying abilities. |
| Turner et al. 2019^100^  United Kingdom | Explore patient and healthcare providers’ experiences of:  (i) residual problems post-TIA/minor stroke; (ii) the impact of TIA/minor stroke on patients’ lives;  (iii) current follow-up care and sources of support. | Descriptive (qualitative) | **1.** No  **2.** Consultation and discussion | **1.** Patients (n=NR)  **2.** NR | NR |
| Wairagkar et al. 2017^101^  United Kingdom | Develop a high dosage, combined language and motor rehabilitation tool for individuals with acquired  brain injury; pilot with three stroke survivors. | Exploratory (methodological) | **1.** No  **2.** Consultation | **1.** Patients (n=2)  Family/caregivers (n=1)  **2.** Patients: both suffered the stroke 11 months prior, suffered from aphasia and upper-limb weakness | - Patient members of the research team indicated satisfaction from a service user’s perspective with the multimodal approach to rehabilitation techniques and enhanced usability for individuals with acquired brain injury. - Suggestions from service users were included in the games developed. - Involvement of service users in the design process, helped increase motivational aspects of the therapy and usability. |
| Webster et al. 2021^102^  United Kingdom | Investigate people with aphasia’s perception of their current reading ability and activities and their feelings about reading. | Exploratory (methodological) | **1.** No  **2.** Consultation | **1.** Patients (n=10)  **2.** Patients: with chronic aphasia, with a mean time post-onset of 88.8± 36.12 months (range 27–143 months, 6 female, mean age of 67.7±11.88 years (range 45–83). There was diversity in terms of years of education, previous occupation, type and severity of aphasia, and reading difficulties. | NR |
| Wilson et al. 2016^103^  United Kingdom | Understand how service factors contribute to delays to specialist assessment following action to seek help from a healthcare professional. | Descriptive (qualitative) | **1.** No  **2.** NR | **1.** Steering group members with patient/carer experience from public representative/support organization (n=NR)  **2.** NR | - The process helped ensure the emergent understanding of issues associated with delays reflected patients’ and general practitioners’ perspectives rather than those of team members. |
| Xian et al. 2015^104^  United States of America | Evaluate the association between warfarin  treatment and longitudinal outcomes after ischemic stroke in the atrial fibrillation population. | Exploratory (cohort) | **1.** No  **2.** A series of focus groups and a qualitative survey | **1.** Patients (n=NR)  Family/caregivers (n=NR)  Public representative/support organization (n=NR)  **2.** Public representative/support organization: stakeholders representing the varied interests of patients, caregivers, physicians, nurses, healthcare systems, policy makers, governmental agencies, and professional societies. | - Patient co-investigators made high value contributions to both the design and the implementation of the study, retaining the rigors of the scientific work. - One of the strengths of the study is the integration of home time, which was a patient centered measure of longitudinal functional outcomes prioritized by stroke survivors and stakeholders. |
| Zhu et al. 2019^105^  China | Development and psychometric evaluation of a scale for patients with stroke, at different stages of their rehabilitation.. | Exploratory (methodological) | **1.** NR  **2.** Interviews, consultation using the Delphi approach | **1.** Patients (n=8)  **2.** Patients: 3 females; mean age of 64.38±16.80 years; mean time since stroke of 15.00±19.89 months; 7 ischemic stroke and 1 both ischemic and hemorrhagic | NR |
