# Supplementary material for: A scoping review of patient and public involvement in empirical stroke research
Source: Int J Stroke. 2024 Jul 31;19(9):962–72. doi: 10.1177/17474930241262638 (PMC11528947; doi:10.1177/17474930241262638)
Supplement: sj-docx-3-wso-10.1177_17474930241262638 – Supplemental material for A scoping review of patient and public involvement in empirical stroke research [file sj-docx-3-wso-10.1177_17474930241262638.docx]

**Supplementary file 3. Adherence to GRIPP2 short form item across included studies.**

| **Item** | **Aim** | **Methods** | **Study results** | **Discussion and conclusions** | **Reflections/critical perspective** |
| --- | --- | --- | --- | --- | --- |
| **Study** | Were the aims of PPI in the study reported? | Were the methods used for PPI in the study described? | Were the results of PPI in the study, including both positive and negative outcomes reported? | Was the extent to which PPI influenced the study (both positive (+) and negative (-)) effects) described? | Did the authors critically reflect on the things that went well (+) and those that did not in PPI (-)? |
| Ainuddin et al. 2021^33^ | Yes | Yes | Yes | Yes (+) | No |
| Allen et al. 2019^34^ | Yes | Yes | No | No | No |
| Andrews et al. 2018^35^ | Yes | Yes | Yes | Yes (+) | Yes (+ and -) |
| Aries et al. 2021^36^ | Yes | Yes | Yes | Yes (+ and -) | Yes (+ and -) |
| Barnfield et al. 2017^37^ | Yes | Yes | Yes | Yes (+ and -) | Yes (+ and -) |
| Bharmal et al. 2016^38^ | Yes | Yes | Yes | Yes (+) | Yes (+ and -) |
| Black et al. 2019^39^ | Yes | Yes | No | No | No |
| Blatchford et al. 2022^40^ | Yes | Yes | No | No | No |
| Bogstrand et al. 2022^41^ | Yes | Yes | Yes | No | No |
| Busetto et al. 2022^42^ | Yes | Yes | Yes | No | No |
| Cadilhac et al. 2016^43^ | No | No | No | No | No |
| Charalambous et al. 2022^44^ | Yes | Yes | Yes | Yes (+) | Yes (+ and -) |
| Clarke et al. 2021^45^ | Yes | Yes | Yes | Yes (+) | No |
| Donetto et al. 2021^46^ | Yes | Yes | Yes | Yes (+) | Yes (+ and -) |
| Eltringham et al. 2019^47^ | Yes | Yes | Yes | Yes (+ and -) | Yes (+ and -) |
| Espinoza et al. 2016^48^ | Yes | Yes | No | No | No |
| Franklin et al. 2017^49^ | Yes | Yes | Yes | Yes (+ and -) | Yes (+ and -) |
| Gesell et al, 2020^50^ | Yes | Yes | Yes | Yes (+ and -) | Yes (+ and -) |
| Golding-Day et al. 2022^51^ | Yes | No | Yes | Yes (+) | No |
| Gustavsson et al. 2020^52^ | Yes | No | No | No | No |
| Hale et al., 2014^53^ | Yes | Yes | Yes | Yes (+) | Yes (+ and -) |
| Hazelton et al. 2022^54^ | Yes | Yes | Yes | Yes (+) | No |
| Hepworth et al. 2019^55^ | Yes | Yes | Yes | Yes (+) | Yes (+) |
| Hepworth et al. 2021^56^ | No | No | No | No | No |
| Heron et al. 2017^57^ | No | No | No | No | No |
| Hinckley et al. 2014^58^ | Yes | Yes | Yes | Yes (+ and -) | Yes (+ and -) |
| Howard et al. 2021^59^ | Yes | Yes | No | No | No |
| Hu et al. 2022^60^ | Yes | Yes | Yes | Yes (+) | No |
| Johnson et al. 2022^61^ | Yes | Yes | Yes | Yes (+) | No |
| Kampling et al. 2020^62^ | Yes | Yes | Yes | Yes (+) | Yes (+ and -) |
| Kearns et al. 2019^63^ | Yes | Yes | Yes | Yes (+ and -) | Yes (+ and -) |
| King et al. 2020^64^ | Yes | Yes | Yes | No | No |
| Kjörk et al. 2022^65^ | Yes | Yes | Yes | Yes (+) | Yes (+ and -) |
| Kwok et al., 2022^66^ | Yes | Yes | Yes | Yes (+) | Yes (+ and -) |
| Kyle et al. 2020^67^ | Yes | Yes | No | No | No |
| Lam et al. 2022^68^ | Yes | Yes | Yes | No | No |
| Langstaff et al. 2014^69^ | No | No | No | No | No |
| Lawrence et al. 2019^70^ | Yes | Yes | Yes | Yes (+) | No |
| Lievesley et al. 2022^71^ | No | No | No | No | No |
| Luo et al. 2015^72^ | Yes | Yes | Yes | Yes (+) | Yes (+) |
| Lynch et al. 2021^73^ | Yes | Yes | Yes | Yes (+) | Yes (+) |
| Manning et al. 2020^74^ | Yes | Yes | Yes | Yes (+) | Yes (+) |
| Manning et al. 2022a^75^ | Yes | Yes | Yes | Yes (+) | Yes (+) |
| Manning et al. 2022b^76^ | Yes | Yes | Yes | No | No |
| Marshall et al. 2020^77^ | Yes | Yes | No | No | No |
| McCormick et al. 2022^78^ | Yes | Yes | Yes | No | Yes (+) |
| McKevitt et al. 2015^79^ | Yes | Yes | Yes | Yes (+) | Yes (+) |
| Mir et al. 2018^80^ | Yes | Yes | Yes | Yes (+) | No |
| Mondal et al., 2022^81^ | Yes | Yes | Yes | No | No |
| Morris et al. 2016^82^ | Yes | Yes | Yes | Yes (+ and -) | Yes (+) |
| Morris et al. 2022^83^ | Yes | Yes | Yes | No | No |
| Nave et al. 2019^84^ | No | Yes | No | No | No |
| Patchick et al. 2015^85^ | Yes | Yes | Yes | Yes (+) | Yes (+) |
| Porat et al. 2019^86^ | Yes | Yes | Yes | Yes (+) | No |
| Prick et al., 2022^87^ | Yes | Yes | Yes | No | No |
| Rai et al. 2021^88^ | Yes | Yes | Yes | Yes (+) | Yes (+ and -) |
| Ramage et al. 2022^89^ | Yes | Yes | Yes | Yes (+ and -) | Yes (+ and -) |
| Rowe et al. 2017^90^ | Yes | Yes | No | No | No |
| Rowe et al. 2022^91^ | Yes | Yes | Yes | No | No |
| Sadler et al. 2016^92^ | Yes | Yes | Yes | Yes (+) | Yes (+) |
| Sadler et al. 2017^93^ | Yes | Yes | Yes | Yes (+ and -) | Yes (+) |
| Shiggins et al., 2022^94^ | Yes | Yes | Yes | Yes (+ and -) | Yes (+ and -) |
| Smith et al. 2018^95^ | No | Yes | No | No | No |
| Solbakken et al., 2022^96^ | Yes | Yes | Yes | No | No |
| Sousa et al. 2019^97^ | Yes | Yes | No | No | No |
| Thayabaranathan et al. 2022^98^ | Yes | Yes | Yes | Yes (+) | Yes (+) |
| Turner et al. 2019^99^ | Yes | Yes | No | No | No |
| Wairagkar et al. 2017^100^ | Yes | Yes | Yes | Yes (+) | Yes (+) |
| Webster et al. 2021^101^ | Yes | Yes | Yes | No | No |
| Wilson et al., 2016^102^ | Yes | Yes | Yes | Yes (+) | No |
| Xian et al. 2015^103^ | Yes | Yes | Yes | Yes (+) | Yes (+) |
| Zhu M et al., 2019^104^ | Yes | Yes | Yes | No | Yes (+ and -) |
